# 3D hierarchical assembly of ultrathin MnO_2_ nanoflakes on silicon nanowires for high performance micro-supercapacitors in Li- doped ionic liquid

**DOI:** 10.1038/srep09771

**Published:** 2015-05-18

**Authors:** Deepak P. Dubal, David Aradilla, Gérard Bidan, Pascal Gentile, Thomas J.S. Schubert, Jan Wimberg, Saïd Sadki, Pedro Gomez-Romero

**Affiliations:** 1 Catalan Institute of Nanoscience and Nanotechnology, CIN2, ICN2 (CSIC-ICN), Campus UAB E-08193, Bellaterra, Barcelona; 2 Univ. Grenoble Alpes, INAC-SPRAM, F-38000 Grenoble, France; 3 CNRS, SPRAM, F-38000 Grenoble, France; 4 CEA, INAC-SPRAM, F-38000 Grenoble, France; 5 Univ. Grenoble Alpes, INAC-DIR, F-38000 Grenoble, France, CEA, INAC-DIR F-38000, Grenoble, France; 6 Univ. Grenoble Alpes, INAC-SP2M-SiNaPs, F-38000 Grenoble, France; 7 CEA, INAC-SP2M-SiNaPS, F-38000 Grenoble, France; 8 IOLITEC Ionic Liquids Technologies GmbH, Salzstrasse 184, 74076, Heilbronn, Germany; 9 Consejo Superior de Investigaciones Científicas (CSIC), Spain

## Abstract

Building of hierarchical core-shell hetero-structures is currently the subject of intensive research in the electrochemical field owing to its potential for making improved electrodes for high-performance micro-supercapacitors. Here we report a novel architecture design of hierarchical MnO_2_@silicon nanowires (MnO_2_@SiNWs) hetero-structures directly supported onto silicon wafer coupled with Li-ion doped 1-Methyl-1-propylpyrrolidinium bis(trifluromethylsulfonyl)imide (PMPyrrBTA) ionic liquids as electrolyte for micro-supercapacitors. A unique 3D mesoporous MnO_2_@SiNWs in Li-ion doped IL electrolyte can be cycled reversibly across a voltage of 2.2 V and exhibits a high areal capacitance of 13 mFcm^−2^. The high conductivity of the SiNWs arrays combined with the large surface area of ultrathin MnO_2_ nanoflakes are responsible for the remarkable performance of these MnO_2_@SiNWs hetero-structures which exhibit high energy density and excellent cycling stability. This combination of hybrid electrode and hybrid electrolyte opens up a novel avenue to design electrode materials for high-performance micro-supercapacitors.

Micro-supercapacitors are miniaturized electrochemical energy storage devices, recently developed, which can offer power densities several orders of magnitude larger than those of conventional batteries and supercapacitors due to their short ion diffusion lengths^1^,^2^,^3^,^4^. Remarkably, such microdevices can be directly integrated into other miniaturized electronic devices such as sensors-actuators or energy-harvesting microsystems providing excellent nano-/micro-scale peak power^5^,^6^,^7^. Recently, great efforts have been devoted to increase the energy and power densities of micro-supercapacitors via the fabrication of nanostructured electroactive materials such as carbide-derived carbon^2^, carbon onions^3^ and the development of thin-film manufacture technologies for example electrochemical polymerization^1^, inkjet printing^8^, and layer-by-layer assembly^9^. In spite of such great advancements, the development of high performance micro-supercapacitors is still a challenge.

The last few years have witnessed a burst of reports on the use of silicon nanowires (SiNW) as electrode materials for micro-supercapacitors due to their fascinating capacitive properties. These include plain silicon nanowires (SiNWs)^10^, as well as doped SiNWs^11^,^12^, silicon carbide nanowires^13^, porous silicon coated with gold^14^,^15^. Moreover, in order to improve the capacitive properties of SiNWs, the development of core-shell nanostructures has been intensively investigated very recently, including NiO/SiNWs^16^,^17^, poly(3,4-ethylenedioxythiophene) (PEDOT)/SiNW^18^ etc. However, due to the intrinsically poor electrical conductivity of metal oxides and the short diffusion distance of electrolytes into pseudocapacitor electrodes, only the surface of electroactive materials can effectively contribute to the total capacitance while the large portion of material underneath the surface could hardly participate in the electrochemical charge storage process, leading to values of areal specific capacitance (ASC) lower than expected. Therefore, it is still a great challenge to boost the electrochemical utilization and ASC of pseudocapacitive materials by rationally designing electrodes with novel microstructures. An emerging attractive concept is to directly grow smart integrated array architectures with the combination of two types of materials and/or nanostructures on conducting substrates as binder-free electrodes for micro-supercapacitors. In this way, many advantages such as multiple accessible electroactive sites, short ion transport pathways, superior electron collection efficiency, and even fascinating synergetic properties are simultaneously achieved to deliver high ASC, sustained cycle life and rate performance.

The overall performance of a supercapacitor depends not only on the electrode materials employed but also on the electrolytes used. Ionic liquids are more expensive than aqueous electrolytes but their relatively superior properties such as high thermal stability, large potential window etc. makes them more promising in supercapacitors. MnO_2_ films with common ionic liquid (IL) electrolyte-based supercapacitors have been investigated with an electrochemical quartz-crystal microbalance (EQCM), X-ray photoemission spectroscopy (XPS)^19^ and *in situ* X-ray absorption spectroscopy (XAS)^20^. It is reported that the most common cations, such as n-butyl-n-methylpyrrolidinium^+^, 1-ethyl-3-methylimidazolium^+^ and 1-butyl-3-methyl-imidazolium^+^, adsorb only onto the electrode surface of the MnO_2_ films and do not penetrate into tunnels within the [MnO_6_] octahedral framework. Thus, a low percentage of Mn in the structure undergoes redox processes with ionic liquid (IL) electrolytes, indicating that ion insertion is correspondingly low. Thus, novel ionic liquid with appropriate cations that enable compensation of the redox reaction during charge and discharge cycles are crucial to improve the capacity performance of MnO_2_ films.

Based on the above considerations, we have fabricated and patented [ref] a unique design of hierarchical MnO_2_@SiNWs core-shell hetero-structure coupled with a novel Li-ion doped ionic liquid as electrolyte, which is based on LiClO_4_ and 1-Methyl-1-propylpyrrolidinium bis(trifluromethylsulfonyl)imide (PMPyrrBTA) for high-performance micro-supercapacitors. In this case the slim SiNWs are the “core” and ultrathin MnO_2_ nanoflakes the “shell” layer. Initially, SiNWs were grown on silicon wafer by chemical vapor deposition (CVD) technique on which subsequent deposition of ultrathin MnO_2_ nanoflakes using chemical bath deposition (CBD) method was carried out. Figure 1 shows a schematic illustration of steps involved in the fabrication of MnO_2_@SiNWs core-shell hetero-structure along with SEM and digital photographs. This MnO_2_@SiNWs device can be cycled reversibly at a high operating voltage of 2.2 V with good capacitance, energy density and excellent cycling stability in a LiClO_4_-PMPyrrBTA IL electrolyte.

## Results

**Figure 2:**
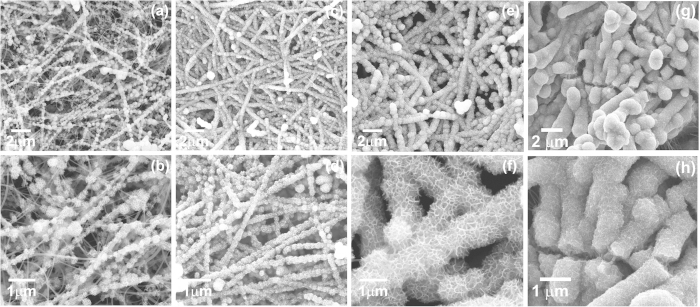
SEM images of the MnO_2_@SiNWs hetero-structures prepared at different deposition times (**a**, **b**) 5 min, (**c**, **d**) 10 min, (**e**, **f**) 15 min and (**g**, **h**) 20 min at two different magnifications, respectively.

Figure 2 presents SEM images of the MnO_2_@SiNWs hetero-structures prepared for different deposition times at two different magnifications. From Fig. 2a,b, one can see that surfaces of SiNWs nanowires are partly covered by MnO_2_ nanoflakes after just 5 minutes of reaction. Yet, many of the nanowires remain uncoated, which indicates an insufficient deposition time. As the reaction time increased to 10 min, almost the whole surfaces of Si nanowires are homogenously covered by ultrathin MnO_2_ nanoflakes (Fig. 2c,d). Further increase in reaction time (15 min) results in SiNWs surfaces covered by highly mesoporous MnO_2_ nanoflakes (Fig. 2e,f), indicative of a sufficiently long reaction time with KMnO_4_. On closer inspection, the individual hierarchical MnO_2_@SiNWs hetero-structure is determined to have a much larger diameter (Fig. 2f), than the pristine Si nanowires. Finally, when the reaction time is 20 min, the resulting hetero-structure is extra thick but less porous and begins to show signs of damage. Indeed, high magnification images show some cracks on the surfaces, not observed in the structures grown during shorter times and therefore most likely due to the over-loading of MnO_2_ on SiNWs (Fig. 2g,h).

**Figure 3:**
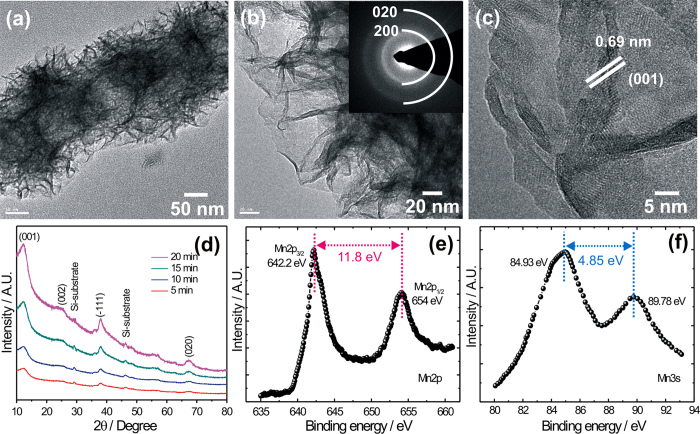
(**a**-**c**) TEM and HRTEM images of the MnO_2_@SiNWs core-shell hetero-structure prepared at the time interval of 15 min, SAED pattern (inset of Fig. 3b) taken from the nanoflake edge. Figure 3 (**d**) XRD patterns of MnO_2_ grown SiNWs at different deposition times. Figure 3 (**e,f**) core level XPS of Mn2p and Mn3s spectra for MnO_2_@SiNWs hybrid materials, respectively.

Figure 3a,b shows TEM images of the MnO_2_@SiNWs core-shell hetero-structure prepared in 15 min time. The surfaces of Si nanowires are uniformly covered by ultrathin nanoflakes (Fig. 3b). The surface of the nanoflake is highly transparent, suggesting very small thickness (~2–5 nm). Further analysis of the SAED pattern (inset of Fig. 3b) taken from the nanoflake edge reveals the formation of birnessite-type polycrystalline MnO_2_^21^,^22^. From the HRTEM image (Fig. 3c), one can clearly see the lattice fringes with an interplanar spacing of 0.69 nm for the two curling nanosheets, which is identified as the characteristic interplanar spacing of the (001) plane of birnessite-type MnO_2_.

The proposed growth mechanism for MnO_2_@SiNWs core-shell hetero-structure is as follows: Initially, MnO^–^_4_ nuclei are produced and adsorbed on surfaces of SiNWs, and form MnO_2_ nuclei upon reduction. With the increase in reaction time, the MnO_2_ nuclei grow and are aggregated and transformed to nanoflakes since thermodynamically, surface energy of individual nanoflakes is high hence they start to self-aggregate (supporting information S1). At the end, the MnO_2_ nanoflake is compact and totally covers the surface of Si nanowires, resulting in the formation of the hierarchical MnO_2_@SiNWs core-shell hetero-structure. Such process is supported by the morphology evolution at different growth stages via tuning the reaction time. In order to determine the crystal phases present in the MnO_2_@SiNWs hetero-structures, X-ray diffraction (XRD) analyses were carried out, as shown in Fig. 3d, where XRD patterns of MnO_2_ grown SiNWs at different deposition times are presented. The diffraction peaks can be indexed as (001), (002), (−111) and (020) corresponding to the birnessite manganese dioxide phase (JCPDS card no. 80–1098, space group of *C*2/m), and confirming the expected formation of MnO_2_. Figure 3e,f shows XPS spectra of the MnO_2_@SiNWs, which are calibrated with reference to C1s peak at 285 eV (supporting information S3). The Mn2p XPS spectrum exhibits two major peaks at binding energies of 642.2 and 654 eV with a spin-energy separation of 11.8 eV (Fig. 3e), in agreement with other reports on MnO_2_ phases^23^. As reported previously^24^, the average oxidation state of Mn in manganese oxides can be determined by the energy separation of Mn3s peaks. The MnO_2_@SiNWs hybrid structures exhibit an energy separation of 4.85 eV for the Mn3s doublet (Fig. 3f), indicating that Mn in the hetero-structure has an oxidation state of Mn(IV).

**Figure 4:**
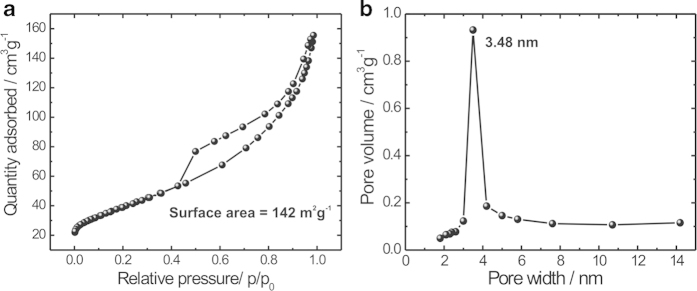
(**a**) Nitrogen adsorption/desorption isotherm of MnO_2_@SiNWs sample (synthesized at 15 min time interval) (**b**) BJH pore size distribution plot of MnO_2_@SiNWs.

To further investigate the surface properties of hierarchical MnO_2_@SiNWs core-shell hetero-structures, we performed Brunnauer-Emmett-Teller (BET) analysis on adsorption isotherms shown in Fig. 4(a). The MnO_2_@SiNWs hybrid structure shows a typical IV- type isotherm with hysteresis loop in a relative pressure (p/p_0_) range of 0.4–1.0, implying the formation of slit-like pores, a type of porosity which can be easily understood as a result of the stacking of MnO_2_ flakes. The BET surface area of the MnO_2_@SiNWs core-shell hetero-structure is calculated to be 142 m^2^g^−1^ which is much higher than plate-like (23–43 m^2^g^−1^) or comparable to nanorods (100–150 m^2^g^−1^), hollow spheres (52–108 m^2^g^−1^) and urchin-like (80–119 m^2^g^−1^) MnO_2_ structures^25^. Figure 4b shows the Barett-Joyner-Halenda (BJH) pore size distribution curve with a distinct maximum centered at ~3.5 nm. This confirms the mesoporous nature of MnO_2_@SiNWs hybrid structure. The mesoporosity of MnO_2_@SiNWs samples results from a combination of internal space of the agglomerated nanoflakes and surface rugosity of the individual nanoflakes. Such type of hierarchical surface morphologies with high surface area and mesoporous nature can enhance electrochemical properties since large pore channels permit rapid electrolyte transport, while the small pores provide more active sites for chemical reactions^26^,^27^.

**Figure 5:**
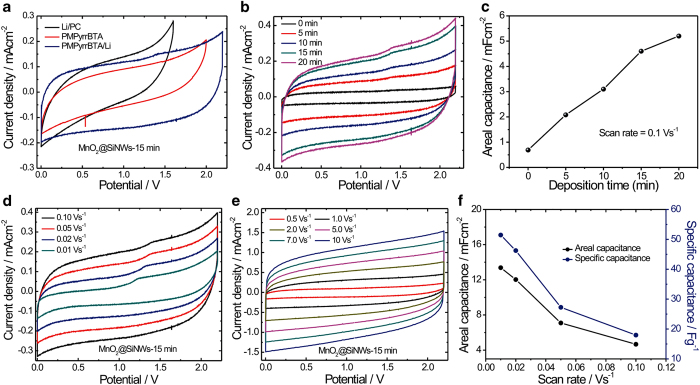
(**a**) CV curves of MnO_2_@SiNWs electrodes in LiClO_4_/propylene carbonate, 1-Methyl-1-propylpyrrolidinium bis(trifluromethylsulfonyl)imide (PMPyrrBTA) and LiClO_4_ doped ionic liquid electrolyte (LiClO_4_-PMPyrrBTA). (**b**) CV curves corresponding to the different MnO_2_ deposition times. (**c**) Variation of areal capacitance with mass loading of MnO_2_ on SiNWs in Li-ion doped ionic liquid electrolyte. (**d**-**e**) CVs curves of MnO_2_@SiNWs (synthesized at 15 min) at different scan rates from 0.01 Vs^−1^ to 10 Vs^−1^. (**f**) Variation of areal and specific capacitance with scan rate of MnO_2_@SiNWs electrodes.

To evaluate the electrochemical performance of the MnO_2_@SiNWs hetero-structure, two-electrode configuration was used in the electrochemical measurements. Figure 5(a) presents the CV curves of MnO_2_@SiNWs electrodes in three different electrolytes, namely i) LiClO_4_/propylene carbonate, ii) 1-Methyl-1-propylpyrrolidinium bis(trifluromethylsulfonyl)imide (PMPyrrBTA) and iii) LiClO_4_ doped ionic liquid electrolyte (LiClO_4_-PMPyrrBTA). Although there are no distinct redox peaks, the shape of CV curve in LiClO_4_/PC electrolyte deviates from the ideal rectangle, implying that the electrode shows faradaic pseudocapacitive nature. However, in PMPyrrBTA, and even more clearly so in Li^+^-doped PMPyrrBTA, the shape of the CV curve is nearly rectangular, indicating that the MnO_2_@SiNWs electrode has satisfactory capacitive behavior in these electrolytes. Furthermore, it is interesting to note that the MnO_2_@SiNWs electrode in Li^+^-doped PMPyrrBTA electrolyte has a substantially larger CV area than in pure PMPyrrBTA. The rectangular CV response, which reflects the pseudocapacitive behavior, was attributed to a continuous and reversible faradaic reaction of the Mn-oxide. Thus, the addition of Li salt in ionic liquid electrolyte significantly increases the electrochemical performance of the MnO_2_@SiNWs electrodes.

In order to investigate the relationship between the deposition time of MnO_2_ and the performances of the devices, we have varied MnO_2_ deposition time from 0 to 20 min. CV curves corresponding to the different MnO_2_ deposition times were measured and are illustrated in Fig. 5(b). All the CV curves were measured at a scan rate of 100 mV/s but show obvious differences. From CV curves, it is seen that, the areal capacitance (related to the area under the CV curves) increases proportionally to deposition time up to 15 min, then gets stabilized at ca. 5.2 mFcm^−2^ as deposition time reaches 20 min Fig. 5(c). We should recall that the microstructural analysis by electron microscopy indicated that the mesoporous structure of MnO_2_ coating on SiNWs started to damage for deposition times longer than 15 min (see SEM micrographs in Fig. 2). Hence, we can conclude that both from a microstructural and an electrochemical point of view, 15 min is an optimal deposition time for MnO_2_.

Next, in order to highlight the merits of this unique hybrid architecture, we tested the hierarchical MnO_2_@SiNWs nanowires as electrodes in symmetrical supercapacitors (two-electrode configuration). For reference, CV curves of MnO_2_ and SiNWs have been tested and in Li-ion doped ionic liquid electrolyte (LiClO_4_-PMPyrrBTA) and shown in supporting information S4. Figure 5(d–e) shows the cyclic voltammograms (CVs) of MnO_2_@SiNWs at different scan rates from 0.01 Vs^−1^ to 10 Vs^−1^ suggesting that the MnO_2_@SiNWs devices can be operated over a wide range of scan rates. Moreover, the CV profiles of the MnO_2_@SiNWs electrodes show the rectangular shape characteristic of capacitive energy storage. This shape remains unchanged as the scan rate increases from 0.01 Vs^−1^ to 10 Vs^−1^, demonstrating good capacitive properties and high-rate capability. Furthermore, the area integrated within the current-potential curves greatly increases for the core-shell arrays as compared with bare SiNWs. This represents a much larger capacity for the hybrid nanowires, which must be attributed to the additional pseudocapacitance provided by the superficial intercalation of Li^+^ions into the thin MnO_2_ flakes that form the nanowires shell. Also, the high scan rate that MnO_2_@SiNWs can achieve (10 Vs^−1^) implies an ultrahigh power density for these unique core-shell hetero-structures. Figure 5(f) shows the variation of areal and specific capacitance with scan rate for MnO_2_@SiNWs electrodes. The highest areal capacitance obtained for the MnO_2_@SiNWs electrode was 13.38 mFcm^−2^ (51.46 Fg^−1^, for mass loading of 0.26 mgcm^−2^) at 0.01 Vs^−1^, which is much higher than the values obtained for the pristine SiNWs (ranging from 0.01–0.05 mFcm^−2^) 11,12,13,14,15 and SiNWs based nanocomposites (for details see supporting information S5). The high areal capacitances we report here are also superior to those found for any of other recently reported hybrid nanostructures. For example MnO_2_/onion like carbon (MnO_2_/OLC) (7.04 mFcm^−2^ at 0.02 mAcm^−2^)^28^, MWCNT/MnO_2_ (2.43 mFcm^−2^ at 0.5 mA)^29^, CNT/MnO_2_ (3.01 mFcm^−2^ at 0.002 mA)^30^, or conducting polymer (PEDOT) coated SiNWs (9 mFcm^−2^ at 0.1 mAcm^−2^)^18^. Moreover, MnO_2_@SiNWs electrodes exhibit a good rate capability with capacity retention of 34.90% of the initial capacitance as the scan rate increases from 0.01 to 0.1 Vs^−1^. Furthermore, it should be remarked that the MnO_2_@SiNWs hybrid symmetric micro-supercapacitors reported here also show better performance in terms of specific capacitance (13.9 mFcm^−2^ at 0.01 Vs^−1^) than interdigitated on-chip micro-supercapacitors based on carbide derived carbon films (e.g. SC: 1.5 mFcm^−2^ at 0.1 Vs^−1^)^31^, or onion-like carbon based micro-supercapacitor electrodes prepared by electrophoretic deposition (e.g. SC: 1.1 mFcm^−2^ at 0.2 Vs^−1^)^32^.

**Figure 6:**
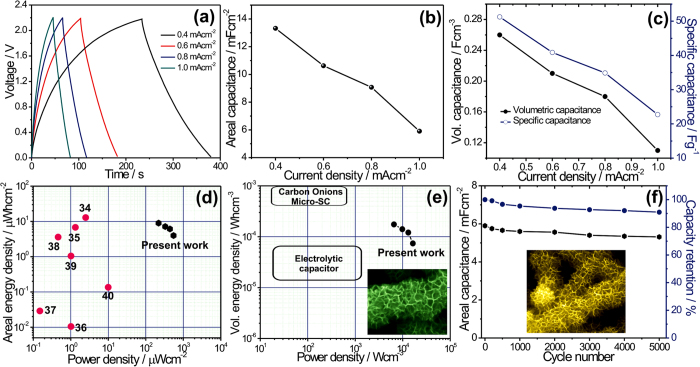
(**a**) Galvanostatic charge-discharge curves of MnO_2_@SiNWs hybrid in Li-ion doped ionic liquid electrolyte at various current densities. (**b**-**c**) The areal, volume and specific capacitances of the MnO_2_@SiNWs symmetric device measured at different current densities. (**d**) Plot of areal energy density versus areal power density of MnO_2_@SiNWs device with comparison to other reported values. (**e**) Plot of volumetric power and energy densities of the MnO_2_@SiNWs device comparing with other energy storage devices. (**f**) Variation of areal capacitance and capacity retention with number of cycles for 5000 cycles.

To further investigate electrochemical performances of the MnO_2_@SiNWs symmetric device, we carried out galvanostatic charge-discharge cycles at various current densities (Fig. 6(a)). The charging and discharging parts of the curves are not perfectly linear which indicates a contribution from the pseudocapacitive mechanism associated to surface intercalation of Li^+^onto MnO_2_. Additionally, a very small *iR* drop (where *i* and *R* represent the current and resistance) for the MnO_2_@SiNWs electrode was observed. This small ohmic drop can be the result of a series of low-resistance connections provided by the solid connection between the silicon substrate and Si nanowires, between the nanowires and MnO_2_ thin sheets as well as the improved ionic conductivity resulting from the addition of a small amount of LiClO_4_ to the PMPyrrBTA-ionic liquid. The areal, volume and specific capacitances of the MnO_2_@SiNWs symmetric device were derived from the discharging curves measured at different current densities and are plotted in Fig. 6(b,c). Remarkably, the MnO_2_@SiNWs device exhibits very high areal capacitance with values up to 13.92 mFcm^−2^ (51 Fg^−1^, 0.26 Fcm^−3^) at a current density of 0.4 mAcm^−2^. These exceptionally good capacitance values can be attributed to the highly porous structure and high specific surface area which facilitate ion transfer and thus enhance redox faradaic reactions and surface adsorption of electrolyte cations. Figure 6(d) compares the areal power and energy densities of the MnO_2_@SiNWs device reported in this work to the values reported for other supercapacitors. The as-fabricated MnO_2_@SiNWs symmetric device features a maximum areal energy density of 9.1 μWhcm^−2^ at a current density of 0.4 mAcm^−2^, which stays in values of 4.02 μWhcm^−2^ (0.07 mWhcm^−3^) at 1 mAcm^−2^, again confirming the excellent rate performance of the MnO_2_@SiNWs hybrid device as shown in Fig. 6(d). Moreover, the obtained maximum volumetric energy density (0.17 mWhcm^−3^) is comparable to carbon/MnO_2_ (0.22 mWhcm^−3^ at 0.02 Acm^−3^)^33^ whereas the areal energy density is considerably higher than SiNWs and carbon based materials^34^. For example, CNT/OMC (1.77 μWhcm^−2^, 0.08 mAcm^−2^)^35^, graphene (0.17 μWhcm^−2^, 0.017 mAcm^−2^)^36^, plastic wire/ZnO nanowires on gold films (0.027 microWcm^−2^, 2 microampere)^37^, pen ink (2.7 μWhcm^−2^ 37, 0.083 mAcm^−2^)^38^, CNT and Ti fibers (0.15 μWhcm^−2^, 0.25 μA)^39^, PANI/stainless steel (0.95 μWhcm^−2^, 0.32 mAcm^−2^)^40^. Moreover, Fig. 6(e) shows volumetric power density versus volumetric energy density for the MnO_2_@SiNWs sample plotted and compared with other energy storage devices such as electrolytic capacitors and carbon onion micro-supercapacitors^41^. It is seen that MnO_2_@SiNWs electrode demonstrates relatively higher energy density than conventional capacitors and higher power density than carbon onion micro-supercapacitors. This observation is quite promising in the context of utilizing MnO_2_@SiNWs samples for fabricating electrodes in supercapacitor devices.

The electrochemical stability of MnO_2_@SiNWs hybrid device was examined by repeated charge-discharge processes at 1 mAcm^−2^. Figure 6(f), shows the evolution of areal capacitance and capacity retention for 5000 cycles. The areal capacitance decreases from 5.9 to 5.31 mFcm^−2^ after 5000 cycles. The overall capacitance loss for MnO_2_@SiNWs device is about 9.1% (90.9% stability) after 5000 cycles. Thus, the unique 3D hierarchical hybrid electrode shows high electrochemical stability for long cycle life applications at high current densities.

## Discussion

As described above, hierarchical ultrathin MnO_2_ nanoflakes can be controllably grown on SiNWs in order to fabricate MnO_2_@SiNWs core-shell hybrid electrodes by a simple solution method followed by a thermal annealing treatment. We would like to discuss here the various reasons why this smartly designed core-shell hetero-structure offers multiple noticeable advantages over previous materials used for micro-supercapacitors applications. For example, (1) well wrapped ultrathin MnO_2_ nanoflakes on SiNWs enable a fast, reversible faradaic reaction, and provide a short ion diffusion path. (2) Moreover, a unique 3D mesoporous structure of MnO_2_ on SiNWs provides a large-area contact for the electrode and electrolyte and enables accommodation of the large volume change and release of the associated strain generated during rapid charge and discharge cycling. (3) Electrically conducting slim SiNWs directly grown on Si wafer serve both as the backbone and electron superhighway for charge storage and delivery. (4) MnO_2_@SiNWs core-shell hetero-structure are strongly supported on Si wafer, avoiding the use of polymer binder/conductive additives and ensuring a sufficiently porous structure, and consequently the “inactive” surface is significantly reduced. (5) Last but not least, The Li^+^doped ionic liquid used here offers additional advantages. Thus, the ionic liquid electrolytes provide a high operating potential of the electrode of 2.2 V whereas LiClO_4_ as the primary ionic working species is reversibly inserted into and out of lattice tunnels between the [MnO_6_] octahedral subunits and cause a large amount of a large amount of the manganese oxide to take part in surface redox reactions.

To conclude, we have developed a facile and cost-effective method to grow hierarchical ultrathin MnO_2_ nanoflakes on SiNWs in order to fabricate MnO_2_@SiNWs hybrid nanocomposite electrodes and demonstrate improved electrochemical performance with Li-ion doped PMPyrrBTA ionic liquid for micro-supercapacitors. By taking advantage of the hybridization of MnO_2_ ultrathin nanoflakes and silicon nanowires (SiNWs), we demonstrate that the device fabricated by the MnO_2_@SiNWs electrodes can be cycled reversibly at a high operating voltage of 2.2 V and exhibits highest areal capacitance of 13 mFcm^−2^. The maximum energy density of 9.1 μWhcm^−2^ (0.17 mWhcm^−3^) and maximum power density of 388 μWcm^−2^ (16 mWcm^−3^) obtained from symmetrical MnO_2_@SiNWs devices with a LiClO_4_-PMPyrrBTA IL electrolyte constitute record-breaking values compared with areal energy and power densities reported in the literature for other micro-supercapacitors. Moreover, it exhibits excellent cycling performance with 91% retention after 5000 cycles. This exciting capacitive behavior is attributed to the unique hierarchical MnO_2_@SiNWs core-shell hybrid structure coupled with Li ion doped IL liquid. This novel double-hybrid approach (with hybridization at the electrode and the electrolyte) has led to the recent filing of a patent^43^ and suddenly adds a novel practical route for the elegant design of high-performance micro-supercapacitors.

## Method

### Fabrication of SiNWs on silicon wafer

Silicon nanowires were fabricated by following the procedure reported elsewhere^42^. SiNWs electrodes with a length of approximately 50 μm and a diameter of 50 nm were grown in a CVD reactor (EasyTube3000 First Nano, a Division of CVD Equipment Corporation) by using the vapor-liquid-solid (VLS) method via gold catalysis on highly doped n-Si (111) substrate. Gold colloids with size of 50 nm were used as catalysts, H_2_ as carrier gas, silane (SiH_4_) as silicon precursor, phosphine (PH_3_) as n-doping gas and HCl as additive gas. The use of HCl has been proven to reduce the gold surface migration and improve the morphology of SiNWs. Prior to the growth, wafer surface was cleaned by successive dipping in acetone, isopropanol and Caro (H_2_SO_4_-H_2_O_2_, 3:1 v/v) solutions in order to remove organic impurities, after that, the substrates were dipped in HF 10% and NH_4_F solution to remove the native oxide layer. Finally, the gold catalyst was deposited on the surface. The deposition was carried out using HF 10% from an aqueous gold colloid solution. The growth was performed at 600 °C, under 6 Torr total pressure, with 40 sccm (standard cubic centimeters) of SiH_4_, 100 sccm of PH_3_ gas (0.2% PH_3_ in H_2_), 100 sccm of HCl gas and 700 sccm of H_2_ as supporting gas. The doping level (dl) of the SiNWs was managed by the pressure ratio: dopant gas/SiH_4_, which was evaluated in previous works (dl: 4 × 10^19^ cm^−3^).

### Growth of ultrathin MnO_2_ nanoflakes on SiNWs

Growth of ultrathin MnO_2_ nanoflakes on SiNWs was carried out by a simple chemical bath deposition (CBD) method. Briefly, 2 millimoles KMnO_4_ was dissolved in 50 ml of deionized water and then 2 ml of hydrochloric acid (98 wt%) was slowly dropped into the above solution. The solution was transparent and free from any precipitate. Then, silicon wafer with pre-deposited SiNWs was immersed in the bath at a temperature of 323 K. After a few minutes, the solution became blurred and a brown precipitate was formed in the bath. During the precipitation an heterogeneous reaction occurred and the deposition of MnO_2_ took place on SiNWs. In order to get uniform coating of MnO_2_, different time intervals such as 5, 10, 15 and 20 min were tested. Finally, MnO_2_@SiNWs substrates were removed, rinsed, and dried in vacuum at 373 K for 2 h.

### Characterization techniques

The surface morphology was studied by scanning electron microscopy (FEI Quanta 650 F Environmental SEM). TEM images were obtained with a field emission gun transmission electron microscope (Tecnai G2 F20 S-TWIN HR(S) TEM, FEI). Crystallographic study was carried out using Panalytical X’pert Pro-MRD instrument (Cu K_α_ radiation and PIXel detector). The X-ray photoelectron spectra (XPS) data were obtained by X-ray photoelectron spectroscopy (XPS, SPECS Germany, PHOIBOS 150). N_2_ adsorption/desorption was determined by Brunauer-Emmett-Teller (BET) measurements using Micromeritics instrument (Data Master V4.00Q, Serial#:2000/2400). Electrochemical characterization of MnO_2_@SiNW hybrid electrodes were carried out in 2-electrode configuration with Biologic VMP3 potentiostat. All samples were measured in the typical two-electrode coin cells with MnO_2_@SiNW hybrid (1 cm × 1 cm) used as both the cathode and anode electrodes. The two electrodes were sandwiched by a PVDF separator and assembled into a coin cell. The material’s mass loading on the sponge is obtained by measuring the weight difference before and after MnO_2_ deposition by using a microbalance. The electrolytes used in this study include 0.1 M of LiClO_4_/propylene carbonate, 1-Methyl-1-propylpyrrolidinium bis(trifluromethylsulfonyl)imide (PMPyrrBTA) (purchased from IOLITEC (Ionic Liquids Technologies GmbH, Germany) and 0.01 M LiClO_4_ doped ionic liquid electrolyte (LiClO_4_-PMPyrrBTA). All cells were assembled and sealed in an Argon-filled glove box.

## Author Contributions

D.P.D. and P.G.R. designed the experiments, analyzed the data and wrote the manuscript. D.A., G.B., P.G. and S.S. carried out synthesis and characterization of silicon nanowires. T.J.S.S. and J.W. provide the ionic liquids. D.P.D. and P.G.R. designed and carried out synthesis and electrochemical measurements of hybrid thin films. To the preparation and reviewing manuscript, all authors contributed equally.

## Additional Information

**How to cite this article**: Dubal, D. P. *et al.* 3D hierarchical assembly of ultrathin MnO_2_ nanoflakes on silicon nanowires for high performance micro-supercapacitors in Li- doped ionic liquid. *Sci. Rep.* **5**, 9771; doi: 10.1038/srep09771 (2015).

## Supplementary Material

Supplementary Information

## Figures and Tables

**Figure 1:**
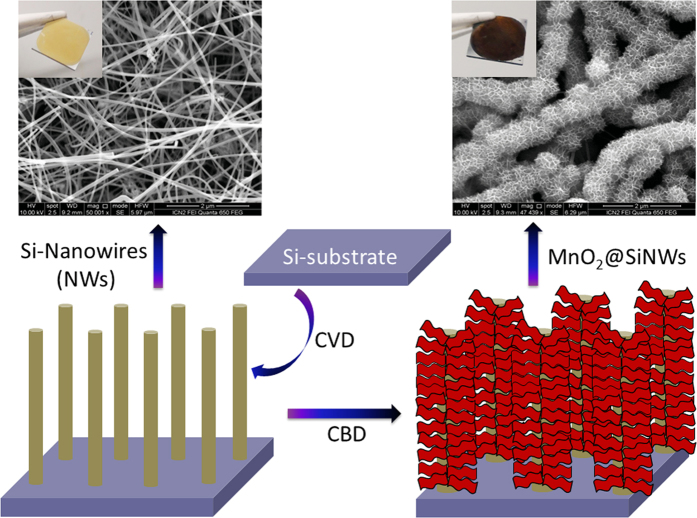
Schematic illustration of steps involved in the fabrication of MnO_2_@SiNWs core-shell hetero-structure along with SEM images and digital photographs of SiNWs and MnO_2_@SiNWs.
