# Supplementary material for: 3D hierarchical assembly of ultrathin MnO2 nanoflakes on silicon nanowires for high performance micro-supercapacitors in Li- doped ionic liquid
Source: Sci Rep. 2015 May 18;5:9771. doi: 10.1038/srep09771 (PMC4434954; doi:10.1038/srep09771)
Supplement: Supplementary Information [file srep09771-s1.doc]

Supporting Information

3D hierarchical assembly of ultrathin MnO_2_ nanoflakes on silicon nanowires for high performance micro-supercapacitors in Li- doped ionic liquid

*Deepak P. Dubal ^a^, David Aradilla ^b, c^, Gérard Bidan ^d^, Pascal Gentile ^b^, Thomas J.S. Schubert ^e^, Jan Wimberg ^e^, Saïd Sadki ^b^, Pedro Gomez-Romero ^a, f^**

^a^Catalan Institute of Nanoscience and Nanotechnology, CIN2, ICN2 (CSIC-ICN), Campus UAB, E-08193 Bellaterra, Barcelona, Spain

^b^LEMOH/ SPrAM/UMR 5819 (CEA, CNRS, UJF), CEA/INAC Grenoble, France

^c^SiNaPS Lab.-SP2M, UMR-ECEA/UJF, CEA/INAC Grenoble, France

^d^INAC/Dir,CEA/INAC Grenoble, 17 rue des Martyrs, 38054 Grenoble, France

^e^IOLITEC Ionic Liquids Technologies GmbH, Salzstrasse 184, 74076 Heilbronn, Germany

^f^Consejo Superior de Investigaciones Científicas (CSIC), Spain

**Supporting Information S1**


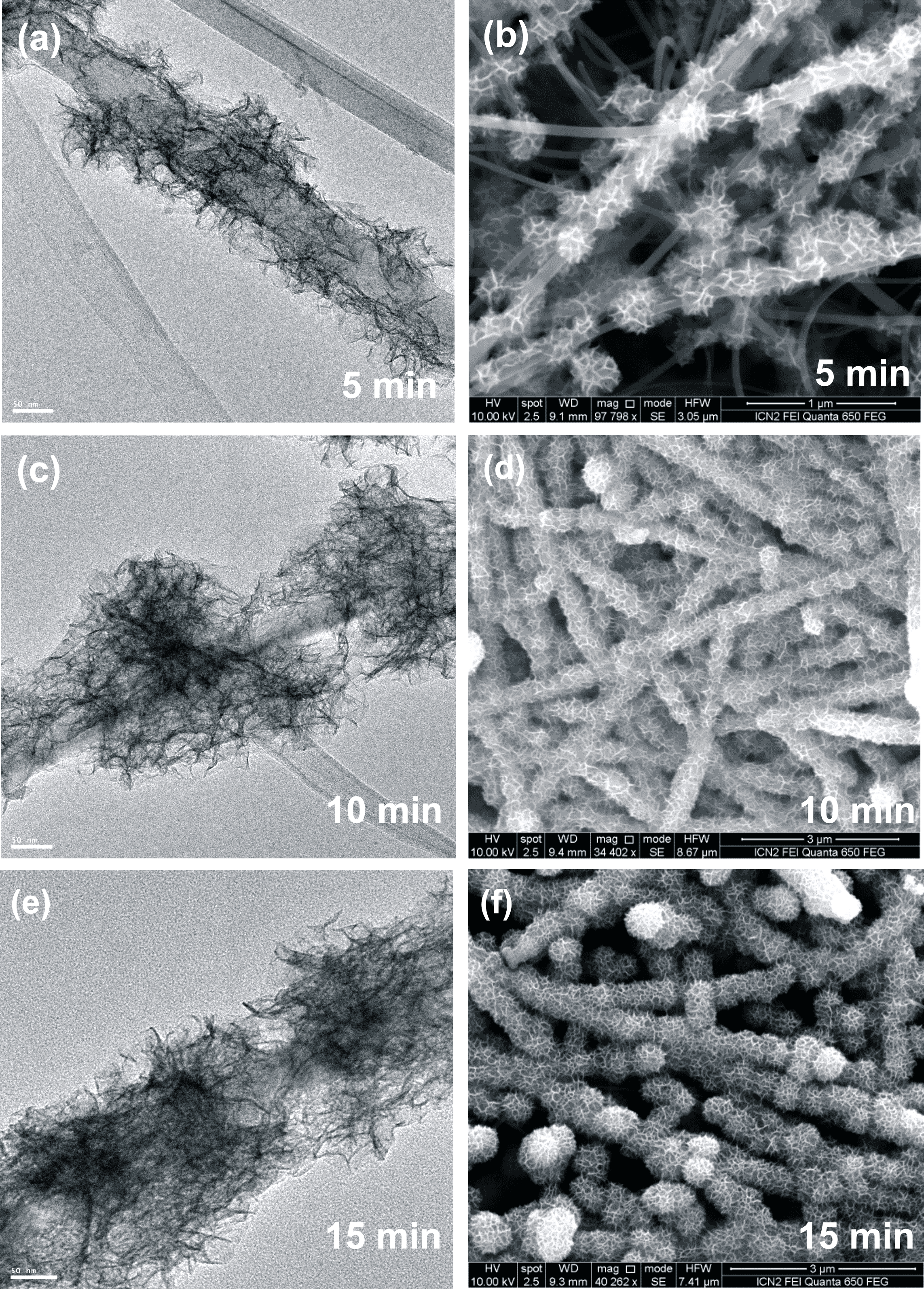


**Figure S1** SEM and TEM images of MnO_2_@SiNWs at different deposition times

**Supporting Information S2**

**
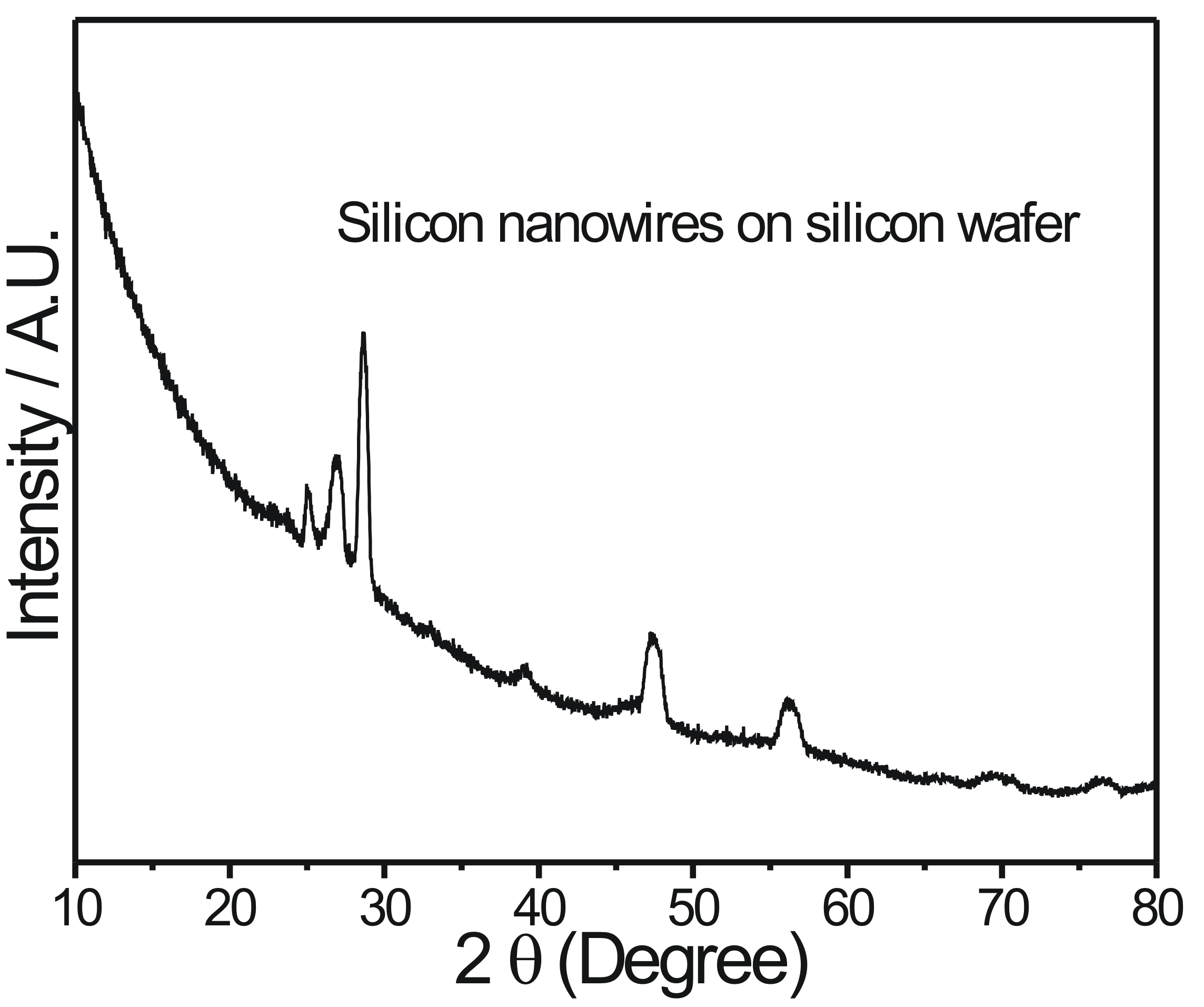
**

**Figure S2** XRD pattern of SiNWs coated on silicon wafer

**Supporting Information S3**


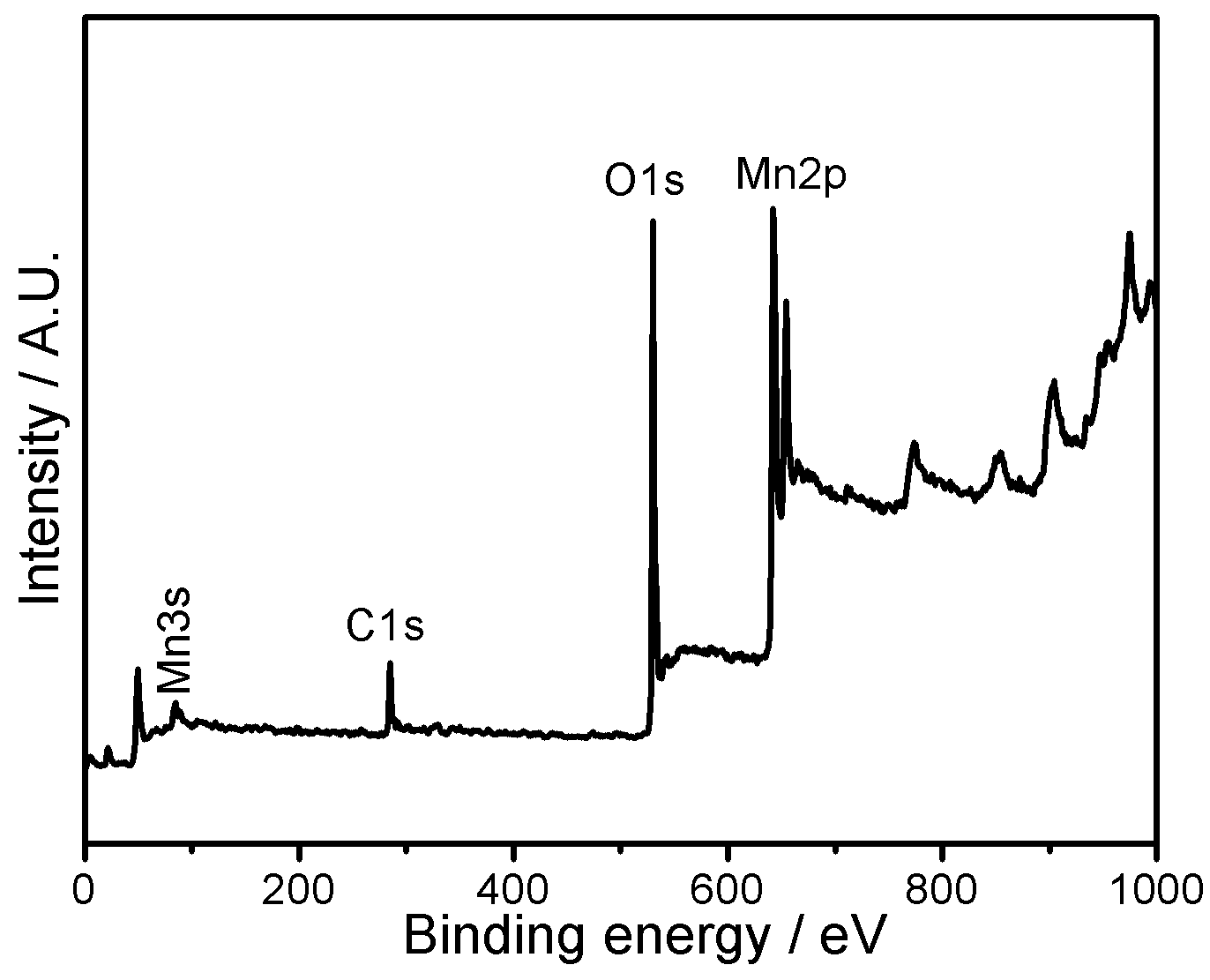


**Figure S3** Full XPS spectrum of MnO_2_@SiNWs synthesized at 15 min deposition time

**Supporting Information S4**

**
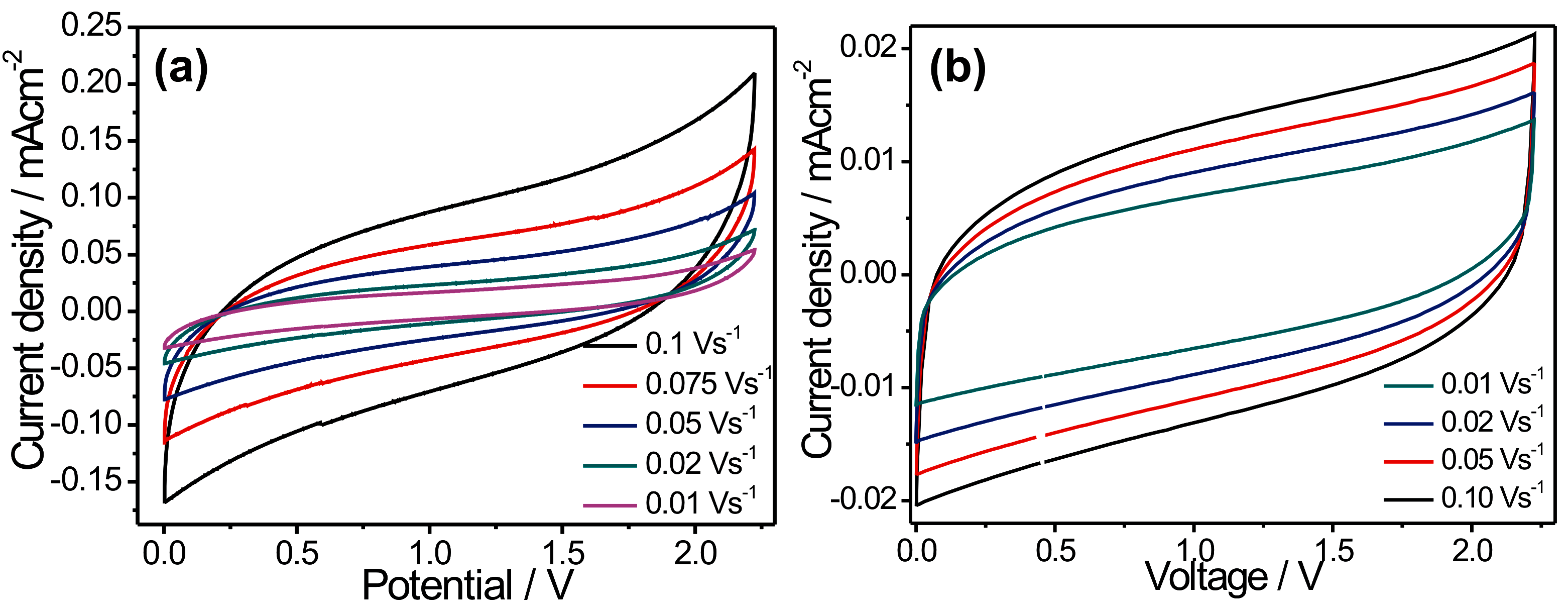
**

**Figure S4** CV curves of symmetric cells of (a) MnO_2_ and (b) SiNWs measured in Li-ion doped ionic liquid electrolyte (LiClO_4_-PMPyrrBTA)

**Supporting Information S5**

**Electrochemical parameters:**

Areal capacitance by cyclic voltammetry,

(1)

where *C*_a_ (mF/cm^2^) is the areal capacitance, *Q* (C) is the average charge during the charging and discharging process, *ΔV* (V) is the potential window and A (cm^2^) is the area of electrode.

Alternatively, areal capacitances of electrodes were measured by galvanostatic charge/discharge method based on the following equation:

(2)

where *C*_a_ (mF/cm^2^) is the areal capacitance, *I* is the constant discharging current, Δt is the discharging time, ΔV (V) is the potential window, and *A* (cm^2^) is the surface area

The volumetric capacitance of the symmetric device calculated from their CVs according to the following equation:

(3)

where *Q* (C) is the average charge during the charging and discharging process is the applied current, V is the volume (cm^3^) of the whole device, *ΔV* (V) is the voltage window. It is worth mentioning that the volumetric capacitances were calculated taking into account the volume of the device stack. This includes the active material, the substrate and the separator with electrolyte.

Alternatively, the cell (device) capacitance (*C*cell) and volumetric capacitance of the electrode (*C*v) was estimated from the slope of the discharge curve using the following equations:

(4)

where *I* is the applied current, V is the volume (cm^3^) of the whole device, *Δt* is the discharging time, *ΔV* (V) is the voltage window.

Volumetric energy density, equivalent series resistance and power density (P, Wcm^-3^) of the devices were obtained from the following equations:

(5)

(6)

where *E* (Wh/cm^3^*)* is the energy density, *C_V_* is the volumetric capacitance and *ΔV* (V) is the voltage window. ESR (Ω) is the internal resistance of the device. P (W/cm^3^) is the power density.
